# Fragility of FDI flows in sub-Saharan Africa region: does the paradox persist?

**DOI:** 10.1186/s43093-023-00184-6

**Published:** 2023-02-02

**Authors:** Folasade Bosede Adegboye, Uchechukwu Emena Okorie

**Affiliations:** 1 Banking & Finance Department, Covenant University, Ota, Nigeria; 2 Fellow Center for Economic Policy and Development Research (CEPDeR), Department of Economics & Development Studies, Covenant University, Ota, Nigeria

**Keywords:** Sub-Saharan African region, Foreign direct investment, Economic development, Fragility, Covid-19

## Abstract

The circumstances of the SSA region regarding the inflow of foreign direct investment (FDI) present a puzzle. In spite of the high rate of return on investment, the inflow of foreign investments keeps eluding the region, and the COVID-19 pandemic even perplexes the flow fragility the more. What factors then determine FDI flows aside from return on investment? Could there be more persuasive relative cost complexes? The study aimed at testing the effects of determining factors that influence FDI flows and their impact on economic development, considering the COVID-19 period. The study used cross-country pooled data from 30 SSA countries collected between 2001 and 2020. The study utilized five panel estimation techniques, namely Pooled Regression, Fixed Effect (FE), Random Effect (RE), Panel Two-Stage Least Square and Differenced Generalized Moments of Method (DGMM). The study found that the inflow of FDI has significant positive impact on economic development in the sub-Saharan African region. It is also ascertained that the outflow of FDI, and political stability has an inverse relationship with economic development. The study recommends that governments of host economies should hence ensure an enabling framework for their economies, so as to improve infrastructure, political stability, and institutional quality, in order to sufficiently encourage the inflow of FDI into the SSA region and make the environment inviting, sustainable, and beneficial for foreign investors and host economies alike.

## Introduction

The COVID-19 pandemic was the worldwide health distress of our generation, and it could be referred to as the biggest struggle the world has borne subsequent to the Second World War [10]. Since its occurrence in Africa in February 2020 [8], the outbreak of the virus has extended not only in Africa but across the globe. The various efforts of countries, as specified by the World Health Organization, to reduce the spread of the disease, primarily movement restrictions and border closures, limited economic activity to the bare minimum [11]. As it was, the health crisis has resulted in additional economic, social, and political crisis primarily for Africa’s developing economies, leaving affected countries with unending consequences [44].

Also, the rebound from the extensive downturn stimulated by the COVID-19 pandemic has been extremely asymmetrical among countries, particularly leaving a good proportion of the low-income countries hanging [11], while high-income economies are rebounding at a faster speed. It is rather shocking to note that only about half of developing countries are likely to achieve their pre-pandemic level of income per capita, worsening their already deplorable impoverishment [46].

The circumstances of Africa regarding the inflow of foreign direct investment (FDI) depict a puzzle. Typically, it is expected that investment will flow from economies with low returns on investment to those with high returns [5, 9, 32]. During the period 2006–2011, the African region had the highest return on investment of more than 11%, compared to 9.1% for Asia, 8.9% for Latin America and the Caribbean, and 7.1% for the global average. In spite of these expectations and opportunities that abound in Africa, the continent’s share of the world’s net FDI has been incessantly low over recent years. Sub-Saharan Africa (SSA) received 1.87% of global net FDI between 2010 and 2016, compared to 26.45% for East Asia and the Pacific, 30.34% for Europe, 17.334% for North Africa, and 13.25% for Latin America and the Caribbean [35].

COVID-19 has gravely disconcerted the global economy and worldwide investment flows. The volume of both FDI projects and capital flows in FDI fell by more than 30% in 2020. FDI projects fell by 33.26% between 2019 and 2020, while investment capital fell by 34% during the same period [40, 42]. This was the steepest drop since 2005, and it fell nearly 20% below the level of foreign investment flows during the global financial crisis of 2009. In particular, FDI inflows to the SSA region fell by 12%, with investment only increasing in a few countries [43]. Countries in West Africa like Nigeria and Togo had increases in inflows of 4.35% and 100%, respectively, while Ghana had a decline of 52%. FDI inflows increased by 3.37% in the Central African sub-region, while they decreased by 16% in the Eastern and Southern sub-regions [43].

The story for 2021, on the other hand, is encouraging, as global FDI increased by 77% over the previous year, surpassing the pre-Covid-19 position. Developed countries experienced the greatest increase, with foreign capital inflows totaling approximately $424 billion at the start of 2021. This is a 300% increase over the unusually low position in 2020 [43]. However, has Africa benefited from this strong bounce back? Developing economies have been said to have benefited immensely from the capital inflow rebound. Is Africa a beneficiary? Surprisingly, FDI into Africa showed a strong rebound, with a 147% increase in 2021 over inflows in 2020. The FDI inflow in 2020 was $39 billion, which increased to $97 billion in 2021. Interestingly, the spike in FDI inflow could be accounted for by a single intra-organization financial trade-off in South Africa, amounting to a 100% increase in FDI inflow in the region in 2021 [43].

Could this mean that the inflow of FDI into the sub-Saharan African region could be relatively increased? Could they be going into sectors that enable African resources to be tapped to their disadvantage? Could they possibly be intra-organizational flows in the form of capital moving within firms and their subsidiaries and not necessarily unencumbered foreign investment? If developing economies are characterized by a high rate of investment return, why then does capital flow uphill? Why do developed economies with lower investment yields have higher investment flows? Evidently, this is a paradox. Does this mean that the higher rate of return on investment for developing economies connotes fragility, uncertainty, and higher relative cost complexity?

Previous studies have examined the impact of FDI on economic growth in the host economies. The impact of FDI on economic development in the SSA region has sparse literature, particularly considering the impact of the global COVID-19 pandemic period and how it affected foreign capital flows. The aim of the study analysis was essentially to determine the impact of FDI, particularly considering the COVID-19 pandemic and how it impacts on the economic development of the host selected thirty (30) SSA countries. The study considered determining socio-economic, institutional, and governance indicators that explain economic development as well as possibly attract an inflow of FDI. The study tested the effect of determining factors on influencing FDI flows and their impact on economic development. The study used cross-country pooled data from 30 SSA countries collected between 2001 and 2020.

## Review of literature

Developing economies in Africa have progressively termed the inflow of foreign investment as a pathway to economic development, generating jobs, harnessing income growth and improving living standards [18]. Many countries open up to international investment inflow and exercise specific approaches to draw in foreign capital. Asiedu [12] determined that several factors that are meant to attract FDI to developing economies, which quite a number of developing economies are in a position to attract foreign capital, work differently for SSA economies. Huge domestic markets’ endowment of natural resources, quality infrastructural facilities, stable consumer price index, and good governance indicators all harness FDI and its productivity [13]. Conversely, the research work of Ayanwale [14] found that openness has a negative association with FDI inflow while infrastructure and investment returns show a positive association with FDI.

Developing economies, including Africa, are characterized by low-income per capita; this explains the limited savings that basically lead to limited domestic investment [6, 29, 30]. The limits of investment can be described, therefore, as inadequate savings that cannot satisfy the requirements of domestic investment. Also, developing economies as well as developed economies have a dearth of skills and technology, which are required production factors, and this hinders investment potential in these countries, hence constraining their capacity to accomplish the desired degree of domestic economic involvement [41].

The research work of Slesman, Baharumshah, & Wohar [39] found that an inflow of capital enhances growth. However, they have been able to ascertain that growth is for economies that have beyond a particular level of institutional quality, and those below this level do not have significant impact or may even be negatively impacted. Likewise, some other research work posits that the dearth of quality institutions is the principal explanation for sub-Saharan Africa’s poor attraction of foreign capital, compared to other developing regions [5, 7, 25]. Dupasquier and Osakwe [23] as well asserted that deficiency in governance was just one of the other determining elements answerable for SSA’s ineptness to draw in foreign investment. Intense research work has gone into the impact of the inflow of FDI on economic growth. Conversely, the impact of economic growth has also been assessed on the inflow of FDI. This creates a possible dual effect on the variables. Premised on panel co-integration and causality tests, Basu, Chakraborty and Reagle [16] put forward that there is a dual-directional causality amid economic growth and FDI in their study carried out on 23 developing countries within the period between 1978 and 1996. Basu et al. [16] assert further that for economies that are reasonably open, causality moves bi-directionally, whereas for economies that are reasonably closed, causality in the long-run mostly moves from growth to FDI [1, 45].

Also, a tangible positive effect on economic growth was determined in a sample of 24 developing economies [33]. However, the study by Carkovic and Levine [19] found that FDI does not have a tangible, positive effect on economic growth in developing countries. This assertion, however, was premised on the implausible theory of the homogeneousness of the lagged variables. Utilizing a disparate panel data framework, FDI has been found to have a positive effect on growth, particularly if the host economy has accomplished a development degree that would assist in reaping the gains of greater productivity [17, 21]. This, however, contradicts the finding of De Mello [22], who asserts that a negative relationship exists between FDI and domestic investment for developed economies.

## Methodology

### Theoretical framework

#### New growth theory

The impact of FDI on economic development has been exceptionally well justified in studies of economics and finance. For example, the new growth theories appraise FDI as a required component for economic growth through its ability to facilitate the transfer of technology, the deluge effect of an increase in domestic savings, skills, and investment, hence, the closing of the limits of capital and foreign exchange, and improving human resource capital and the quality of institutions [6, 20, 31]. Hymer [28] asserts that developing economies are characterized by low-income per capita and, hence, high investment returns. It is explained that there is an inverse relationship between income per capita and return on investment. These concepts, hence, acknowledge the fact that FDI is an essential pathway through which the developed economies can reach out to the developing parts of the world. There is also a significant intersection and relationship between FDI and trade globally. Variations in determining factors, such as the quality of institutions, trade policies, and human resource capital of host economies, provide a compelling justification for certain disparities in FDI and development outcomes.

Subsequent to several reviewed theories, this study is premise on the new growth theory. The motive for embracing the new growth theory as a standard theory is hinged on the evidence that the method is focused on the wants of man as well as the indefinite needs in advancing development economically. It also asserts that advancement in technology and innovation most times do not originate unexpectedly. Comparatively, it depends on the frequency of advancement in technology, the infrastructural demands and the extent of persistence. The extent of sufficiency, advancement of technology, enhancement of infrastructure and an enabling business environment would advance economic development [3, 27]. According to Hulten [27], new growth theories are premised majorly on more recent hypotheses that the marginal product of capital is more constant relative to the decreasing level as presented in the Neoclassical growth model. Mostly, in new growth theories, capital includes determinants of investment, INFSR, PCIG, PSAV, GEFF, and human development required to achieve desired economic development.

Therefore, the implicit function of the model is as follows:

1 $$ {\text{HDRI }} = f \, \left( {{\text{FDII}},\;{\text{FDIO}},\;{\text{GEFF}},\;{\text{INFR}},\;{\text{PCIG}},\;{\text{PSAV}}} \right) $$

The model in Eq. (1) is explicitly represented as

2 $$ {\text{HDRI}}_{it} = \, \beta_{0} + \, \beta_{1} {\text{FDII}}_{it} + \, \beta_{2} {\text{FDIO}}_{it} + \, \beta_{3} {\text{GEFF}}_{it} + \, \beta_{4} {\text{INFR}}_{it} + \, \beta_{5} {\text{PCIG}}_{it} + \, \beta_{6} {\text{PSAV}}_{it} + \varepsilon_{it} $$

From Eq. (2);

HDRI means the human development index, which is the dependent variable and proxy for economic development.

FDII represents FDI net inflow.

FDIO connotes FDI outflow.

GEFF captures government effectiveness.

INFR represents infrastructure.

PSAV represents political stability and absence of violence, and.

PCIG represents per capita income growth.

β_0_ is the constant term.

β_1 …_ β_6_ are the slope coefficients of the model.

From the model, ‘i’ and ‘t’ represent entities as well as time in respective order. Entities in this research work represent the 30 SSA countries selected from four regions which make up the region, within the period 2001 to 2020.

(Theoretical relationship between variables used in the model and FDI).

The world all round, economies are moving in the direction of attaining economic development, toward achieving the United Nation (UN) SDGs well ahead of 2030 [24]. Attaining sustainable development can be achieved by countries in the SSA region by establishing proficient human capital and ensuring valuable institutional quality [37]. This is attainable by ensuring that factors that determine foreign capital inflows (FDII), foreign capital outflow (FDIO), as well as enable a workable business environment are embraced. Hence, establishing more infrastructure (INFR), ensuring politically stable environment without violence (PSAV), government effectiveness (GEFF), aids investment to flow in and advance toward sustainable growth, in order to attract and retain more foreign investors and thereby enhance domestic investment as income per capita (PCIG) is improved [5, 24]. In SSA countries, the degree of human development index (HDI), attainable by creating a resilient institutional environment to attract FDI inflow in order to attain development economically, has minimal resource in the literature, particularly in accounting for the impact of the Covid-19 pandemic; therefore, this informs the rationale for this research work.

(Explanatory information about the development of variables in African countries (SSA)).

This empirical model of the research work is similar to the previous research work of Adegboye et al. [5]. The study aims at determining how factors that determine foreign capital flows, such as government effectiveness, infrastructure, and a politically stable environment without violence, can attract investment inflow to advance sustainable growth and economic development for the SSA countries. The main motivation for the selected variables is premised on the validity of the requirements for economic growth and its attainment in the SSA region, institutional quality; investment components; FDI flows; and infrastructure should be present in the model. It is the gap in the literature that this study is aimed at filling while accounting for the period of the COVID-19 global pandemic.

#### Empirical procedure

In the first instance, stationarity of the data observation was verified using the Levin, Lin and Chu approach to panel unit root testing. The test was conducted at level and first differencing of the data series to attain stationary state suitable for credible data estimation and analysis for sustainable policy recommendations. After establishing the order of integration of the data observations, a panel approach for regression involving pooled regression, fixed and random effect regression was utilized to establish the extent and nature of the relationship between the dependent variable Human Development Report Index (HDRI) and the exogenous variables in the model. As the name implies the pooled regression estimates, the data set as a pooled observation not accounting for the countries specific heterogeneous factors while the fixed effect technique provides us with the opportunity of taking cognizance of the time invariant characteristic and individual effect of the country specific factors.

Random effect assumes the idiosyncratic errors associated with the group specific variables are randomly distributed and non-systematic that could constitute significant error bias. Hence, the study employs the Hausman test (Table 5) in determining whether there is a significant difference between the consistent fixed effect estimates and the efficient random effect estimate [26]. In the presence of a significant difference, the consistent fixed effect estimate was selected in this study. The study further employed the two-stage least square approach involving instrumental variable (IVs) estimation to control for possible endogeneity bias of the fixed effect result as explained in the result section. Further estimation process higher precision and with robustness check was employed to account for possible endogeneity arising from endogenous regressors and inherent country fixed effects with the introduction of difference generalized moment method (DGMM) suitable for dynamic panel data estimation and cross-sectional observation with higher cross-sectional dimension.

## Results and discussion

Given the importance of ascertaining the extent of the stochastic and deterministic trend properties of the data considered in this study, the panel unit root test with Levin, Lin and Chu (LLC) was conducted and the result displayed in Table 1 of the analysis section.

**Table 1:** Panel Unit Root Result. *Source*: Computation from World Development Indicator 2022 using E-view

Variable	LLC @levels	Remark	LLC @ first Difference	Remark
FDII	3.50647***	I(0)	NA	NA
FDIO	6.24107***	I(0)	NA	NA
INFR	1.82054	Non stationary	12.3092***	I(1)
PCIG	1.85249**	I(0)	NA	NA
PSAV	0.37578	Non stationary	9.79016***	I(1)
GEFF	− 0.01736	Non stationary	− 6.50875***	I(1)
HDRI	7.84198***	I(0)	NA	NA

*, **, *** denote stationarity at 10%, 5% and 1% significance level. LLC represents Levi-Lin-Chu unit root test. NA: Not applicable; I (0): Integrated of order zero; I (1): Integrated of order 1

The stationarity of the data series was tested using LLC as shown in Table 1. The result indicates that the variables foreign direct inward (FDII), Foreign direct outward (FDIO), Per capita income growth (PCIG) and human development index (HDRI) were stationary at levels which suggests they were integrated of order zero I(1) while other variables comprising of infrastructure(INFR), political stability-absence of violence (PSAV) and government effectiveness(GEFF) were not stationary at level and were differenced once to attain stationary process and were observed to be integrated of order 1. This shows that all the variables in the human development index model were integrated to order zero and 1 at 1% and 5% level of significance. This implies that the variables are stationary and within the unit circle. Hence, they could easily revert to the equilibrium state in the incidence of external shocks to the system and the results of the parameter estimates would be valid for sustainable policy recommendations.

The descriptive statistics in Table 2 shows the mean, median, maximum and minimum range of the variables. It also portrays the pattern and direction of the variables’ distribution using the standard deviation, skewness, kurtosis and Jarque–Bera normality statistics. The mean values indicate infrastructure (INFR) has the highest average score among the entire variables used by the model. This is further depicted in the median, maximum and minimum scoring of the variables. In terms of the standard deviation measure, it observed that infrastructural spending among the Sub-Saharan countries exhibited the highest degree of variability, positively skewed toward the right with highest concentration toward the top. Conversely government effectiveness (GEFF) witnessed the lowest ranking in respect to its mean score, median, range, negative skewness, concentration toward the peak and in terms of its variability with the exception of the COV19 dummy. More insight from the descriptive result shows most of the variables were associated with asymmetric distribution except for political stability absence of violence measure. This also affirms to the pertinence of the test for stationarity of the variables as indicated in Table 1.

**Table 2:** Descriptive statistics. *Source*: Computation from World Development Indicator 2022 using E-view

	HDRI	COVDUM	FDII	FDIO	GEFF	INFR	PCIG	PSAV
Mean	0.487683	0.100000	4.558228	0.966760	0.298970	37.72366	1.540673	0.665561
Median	0.478000	0.000000	2.240929	0.090387	0.250000	36.00000	1.729956	0.670000
Maximum	0.709000	1.000000	103.3374	75.99954	0.630000	95.53354	28.67600	0.880000
Minimum	0.298000	0.000000	− 6.369877	− 32.23268	0.000000	1.300314	− 31.33308	0.420000
Std. Dev	0.087192	0.300250	9.118275	6.059571	0.194471	24.02206	4.697645	0.098246
Skewness	0.276042	2.666667	5.918041	7.053503	− 0.202055	0.449686	− 0.720645	− 0.106707
Kurtosis	2.805194	8.111111	50.14644	72.84994	2.057410	2.236977	11.44815	2.664887
Jarque–Bera	8.111664	1364.198	59,072.00	107,484.8	19.15118	32.16862	1836.213	2.874107
Probability	0.017321	0.000000	0.000000	0.000000	0.000069	0.000000	0.000000	0.237627

The correlation matrix in Table 3 indicates no strong level of correlation among the variables used in the model analysis. This further suggests that there is no evidence of multi-collinearity in the model. The detailed analysis of the degree of correlation effect within the exogenous variables indicates the highest coefficient of 0.576 between FDI inward and outward but less than 0.80, which can be considered a high degree of multi-collinearity. This study therefore concludes that there is an absence of a high level of interdependency among the explanatory variables considered by the study model.

**Table 3:** Correlation matrix. *Source*: Computation from World Development Indicator 2022 using E-view

	HDRI	COVDUM	FDII	FDIO	GEFF	INFR	PCIG	PSAV
HDRI	1	0.148557	− 0.04991	− 0.00618	0.435546	0.770921	− 0.12535	0.18027
COVDUM	0.148557	1	− 0.02396	− 0.03244	0.067687	0.114909	− 0.08093	− 0.07128
FDII	− 0.04991	− 0.02396	1	0.577697	− 0.18386	− 0.19022	0.04742	0.098181
FDIO	− 0.00618	− 0.03244	0.577697	1	− 0.1853	− 0.08603	0.048057	− 0.02104
GEFF	0.435546	0.067687	− 0.18386	− 0.1853	1	0.303011	− 0.01463	0.34944
INFR	0.770921	0.114909	− 0.19022	− 0.08603	0.303011	1	− 0.09359	0.113875
PCIG	− 0.12535	− 0.08093	0.04742	0.048057	− 0.01463	− 0.09359	1	0.037759
PSAV	0.18027	− 0.07128	0.098181	− 0.02104	0.34944	0.113875	0.037759	1

In order to ensure the efficiency and reliability of the parameter estimates, the study utilized five panel estimation techniques involving pooled regression, Fixed Effect (FE), Random Effect (RE), Panel Two-Stage Least Square (PTSLS) and Differenced Generalized Moments of Method (DGMM). In the DGMM, the differenced instruments of GMM were employed to account for the serial correlation as a result of the endogenous regressors that could constitute a potential source of endogeneity in the estimated model. The pooled regression was conducted by pooling the entire data sample across the 30 countries over the 20 years’ period. The result of the pooled estimates shows that COV19 dummy, FDII and FDIO exerted significant negative influence on HDRI at 1%, 10% and 5% significance level. PCIG indicates significant positive relationship with HDRI at 5% level, while PSAV is negatively and significantly related with HDRI at 5% level with a magnitude impact of − 0.02%. Hence, the pooled estimates show that all the variables except for GEFF and INFR indicate significant effect on HDRI while PCIG had a significant direct impact on HDRI.

The Hausman test (Table 5) of statistical significance was employed to examine whether there is an existence of a significant difference between the consistent fixed effect and the efficient random effect estimates. The result of the test shows that the idiosyncratic errors were not by chance and hence constitute a significant disturbance to the system which could bias the random effects estimates. Thus, the fixed effect estimates become more appropriate to be interpreted.

However, the random effect result shows a significant and positive relationship between COV19 dummy, FDII and HDRI at a 10% level of significance, while GEFF and INFR indicate a significant positive impact of 0.08 and 0.003 on HDRI at a 1% significance level. On the contrary, PSAV is observed to be negatively related to HDRI with a magnitude impact of 0.12%, considered at a percent level.

Given the Hausman selection, this study result focuses more on fixed effect (FE), which accounts for the time invariant heterogeneity in respect of the country’s specific characteristics. The result shows that COVDUM and PSAV are negatively and significantly related to HDRI at the 5% significance level, with a magnitude impact of − 0.002 and − 0.011, respectively. At the 1% significance level, FDII and PCIG had a positive and significant influence on HDRI, whereas PSAV had a negative and significant influence on HDRI with a − 0.011 impact. The FE model estimates indicate that all variables, with the exemption of GEFF and INFR, had a significant impact on HDRI. However, given the trace of endogeneity issues still associated with the fixed and random effect models, this study went further to employ an instrumental variable approach in the estimation process to address this issue, and the result is as present in the last column of Table 4.

**Table 4:** Panel results. *Source*: Computation from World Development Indicator 2022 using E-view

Variables	Pooled	RE	FE	PTSLS	DGMM
FDII	− 0.00007*	0.000381*	0.0000886***	0.0000153	0.000161
FDIO	− 0.00009**	− 0.000125	− 0.000123***	− 0.000148***	− 0.000277***
GEFF	0.011502	0.080723***	0.008457	− 0.005317	0.002208***
INFR	− 0.00007	0.003179***	− 0.0000435	0.000287	− 0.0000241
PCIG	0.000148**	0.000462	0.000176***	− 0.000467***	0.000591***
PSAV	− 0.015766**	− 0.118112***	− 0.010718**	− 0.031289*	− 0.008283***
COVDUM	− 0.00320***	0.010692*	− 0.001927**	− 0.000497	****
Constant	1.14831***	0.420805***	0.776496***	0.660729	****
*Diagnostic Test*					
*F*-Statistics	14,589.47	58.28	7469.89	7050.460	****
Probability > *F*	0.000	0.0000	0.000	0.000	****
*R*-Squared	0.99724	0.53753	0.99833	0.998141	****
Adj. *R*-squared	0.99717	0.52830	0.99815	0.997939	****
Durbin Watson	1.12557	0.26708	1.41861	1.855672	****
Instrument rank				34	25
Prob (*J*-statistic)				0.464440	0.200015
*J*-statistic					20.94825
A&B S. C Test					0.611947
Probability					0.5406

*, **, ***Denote significance level at 10%, 5% and 1% while ****indicates Not Applicable

A&B S.C Test-Arrellano-Bond Serial Correlation Test

The Panel Two-Stage Least Square (PTSLS) regression was further employed in controlling for endogeneity in the model estimation with the introduction of instrumental variables (IVs) that are correlated with the regressors but uncorrelated with the error term. This is evidenced by Durbin and Watson’s result (1.9), which indicates the absence of serial autocorrelation in the model. Also, the instrument rank and its related probability of the *J*-statistics (*P*-value > 0.01) further attest to the statistical relevance of the instrumental variables to the model. The evidence from the PTSLS estimates shows that COVDUM had a significant negative impact on HDRI at a 5% level, while FDIO and PCIG estimates negatively and significantly influenced HDRI at a 1% level. In contrast, FDII, GEFF, and INFR apparently are insignificant in explaining human capital development, though positively related to it (Table 5).

**Table 5:** Hausman Test of Significance. *Source*: Computation from World Development Indicator 2022 using E-view

Correlated Random Effects—Hausman Test				
Test cross-sectional random effects				
Test Summary	Chi-Sq. Statistic	Chi-Sq. d f	*P*-value	
Cross-sectional random	35.399569	7	0.0000	

df: Degree of freedom

Hence, from the most statistically significant selected model (FE), in this study, it is observed that the dummy variable for COVID 19, foreign direct investment outflow, political stability, and absence of violence were significantly and inversely related to the human development index. This is suggestive of the fact that the negative impact of the global pandemic is also significantly reflected in human development. The evidence of the negative influence of FDI outflow is an indication that the outflow of foreign capital worsens economic development. Also, for per capita income growth, it shows an indication of further slide into a higher poverty index, hence, the inverse effect on human development in the sub-Saharan Africa (SSA) region. This could be explained by the fact that FDI outflow could have been used to develop domestic investment in order to have sustainable development. Likewise, the development of labor through effective collaboration between foreign and indigenous expertise workers could be impaired due to FDI outflow.

An increase in PCIG has positively and significantly contributed to human development. Political stability and absence of violence, in addition to FDI outflow from African countries, consequently reflect an adverse effect on human development, especially among African developing economies within the SSA context. The F-statistics (14589.47; 58.28; 7469.89; and 7050.46) with *P*-values less than 1% significance level indicate that the respective pooled, random effect, fixed effect, and two-stage least square models are statistically significant with the data well-fitting the model. The results of the R-squared and its adjusted components show a high explanatory strength of the exogenous variables in explaining the dependent variable-human development index for the respective estimation techniques.

The estimated GMM result further confirmed that FDI outflow and political uncertainty and violence have significantly retarded human development in SSA economies, while government effectiveness and higher earnings in the form of increased per capita income have exerted a significant positive influence on human development. This further reinforces the idea that good governance and an improved standard of living play a significant role in human capital development. The diagnostic test of the reliability of the instruments was further confirmed to be statistically significant with the *P*-value of the *J*-statistic (0.20 > 0.05). The source of endogeneity was adequately addressed using the Arellano-Bond serial correlation test with a *P*-value greater than 5% significance level and the study’s acceptance of the null hypothesis of serial correlation.

## Conclusions and policy implications

This study empirically tested the effect of determining factors that influence FDI flows and their impact on economic development, particularly considering the global COVID-19 pandemic. The research work adopted cross-country pooled data from 30 SSA countries within the period 2001 to 2020. The FE and DGMM tests confirm that the inflow of FDI and per capita income growth have a significant positive impact on economic development in the sub-Saharan African region. This affirms the findings of Adedeji and Ahuru [2], Obadan [34], Okwu et al [36], whose work asserts that FDI inflow stimulates growth in the SSA region. It further confirms that institutional and governance indicators determine the inflow of FDI into SSA countries. This affirms the submissions of Asiedu [13], Baltabaev [15], Sarode [38], and Adegboye et al. [4]. It further asserts that the outflow of FDI, political stability, and the absence of violence have an inverse relationship with economic development.

It was observed that though FDI inflow is significant, the impact is minimal for the selected host SSA countries. This implies that the inflow of foreign capital is possibly going into non-investment beneficial sectors or sectors of the economy that are not bringing about the development of domestic investment, hence no economic development. This is supported by the argument Adegboye [3] and Adegboye et al. [6] that the receiving sector of FDI in host economies determines the extent of development derived. Though the growth in income is also positive, the extent of its impact is minimal as well, as people are going further into poverty with the incessant rise in prices, thus making domestic investment elusive. Also, the presence of violence in the region and political instability are evidently showing how averse the business environment is to attracting foreign capital, hence, the inverse impact on economic development.

The governments of host economies should hence ensure an enabling framework for their economies, so as to improve infrastructure, political stability, and institutional quality, in order to sufficiently encourage the inflow of FDI into the SSA region. The region has the potential to attract foreign investment with the right enabling framework and institutional quality. It would attract FDI just as it is increasingly flowing into other developing regions of the world. The paradox of the uphill flow of capital truly subsists and foreign capital still does not flow sufficiently into the SSA region in spite of the enormous opportunities for high return on investment. The fragility of FDI in the SSA region explains why it defies what obtains in other developing regions globally. An enabling framework could make the environment inviting, sustainable, and beneficial for the foreign investor and the host economies alike.

## Data Availability

Yes. Data are available.

## Abbreviations

AU: African Union
COVID-19: Coronavirus Disease 2019
COVDUM: Covid-19 Dummy variable
FDI: Foreign Direct Investment
FDII: Foreign Direct Investment Inflow
FDIO: Foreign Direct Investment Outflow
FE: Fixed effect
GEFF: Government effectiveness
HDI: Human development index
HDRI: Human development report index
INFR: Infrastructure
LLC: Levin, Lin & Chu
PCIG: Per Capita Income Growth
PSAV: Political stability & absence of violence
PTSLS: Panel two-stage least square
RE: Random effect
SDGs: Sustainable development goals
SSA: Sub-Saharan Africa
UN: United Nations
UNCTAD: United Nations Conference on Trade and Development
